# A mutant p53/Hif1α/miR-30d axis reprograms the secretory pathway promoting the release of a prometastatic secretome

**DOI:** 10.15698/cst2020.11.235

**Published:** 2020-10-05

**Authors:** Valeria Capaci, Fiamma Mantovani, Giannino Del Sal

**Affiliations:** 1 Dipartimento di Scienze della Vita, Università degli Studi di Trieste – Trieste (TS), 34127 – Italy.; 2 Cancer Cell Signalling, International Centre for Genetic Engineering and Biotechnology-Trieste (TS), 34149 - Italy.; 3 Fondazione Istituto FIRC di Oncologia Molecolare (IFOM) – Milan (MI), 20139 – Italy.

**Keywords:** mutant p53, miR-30d, Golgi Apparatus, secretory machinery, microenvironment

## Abstract

*TP53* missense mutations are frequent driver events during tumorigenesis. The majority of *TP53* mutations are missense and occur within the DNA binding domain of p53, leading to expression of mutant p53 (mut-p53) proteins that not only lose the tumor suppressive functions of the wild-type (wt-p53) form, but can also acquire novel oncogenic features fostering tumor growth, metastasis and chemoresistance. Mut-p53 affects fundamental cellular pathways and functions through different mechanisms, a major one being the alteration of gene expression. In our recent work (Capaci *et al.*, 2020, Nat Commun) we found that mut-p53, via miR-30d, modifies structure and function of the Golgi apparatus (GA) and induces increased rate of trafficking. This culminates in the release of a pro-malignant secretome, which is capable of remodeling the tumor microenvironment (TME), to increase stiffness of the extracellular matrix (ECM), favouring metastatic colonization, as shown by cell-based assays and experiments of metastatic niche preconditioning in mouse xenograft models. This study provides new insights into the mechanisms by which mut-p53, through induction of non-coding RNAs, can exert pro-tumorigenic functions in a non-cell-autonomous fashion, and highlights potential non-invasive biomarkers and therapeutic targets to treat tumors harboring mut-p53 (Figure 1).

## MUT-p53 AS A DRIVER OF A MALIGNANT SECRETOME

p53 mutants can promote tumorigenesis by affecting fundamental cellular pathways and functions, and by modulating the interplay of cancer cells with the TME. The role of mut-p53 in this process is dual. On one hand, previous reports provided evidence that mut-p53 directly regulates transcription of several secreted proteins that promote pro-invasive changes of the microenvironment. These include soluble mediators (e.g. growth factors, chemokines and cytokines) as well as ECM components. On the other hand, in this work we define a new paradigm by which mut-p53 acts as a driver of a malignant secretome, that is by enhancing the whole secretion process via inducing structural changes of the entire GA. The mut-p53-dependent secretome affects both transformed and stromal cell populations in a paracrine fashion, promoting migration, ECM deposition and remodeling, stromal neo-vascularization, and CAF activation both at the primary and secondary tumor site, accelerating the timing of metastasis formation *in vivo*. All these aspects are achieved via induction of miR-30d by mut-p53 in concert with the hypoxia-responsive factor HIF1α. Importantly, mut-p53 interacts with HIF1α not only under hypoxic conditions, as previously reported, but also under normoxia, suggesting that mut-p53 might induce HIF1α-dependent responses in cancers independently of oxygen availability. Conversely, activated HIF1α can induce miR-30d expression also in cells that do not express mut-p53. This implies that HIF1α-inducing stimuli can modify GA structure and secretory trafficking (via miR-30d) also in tumors lacking missense *TP53* mutations, however, if present mut-p53 potentiates the efficiency of HIF1α-induced molecular changes.

**Figure 1:**
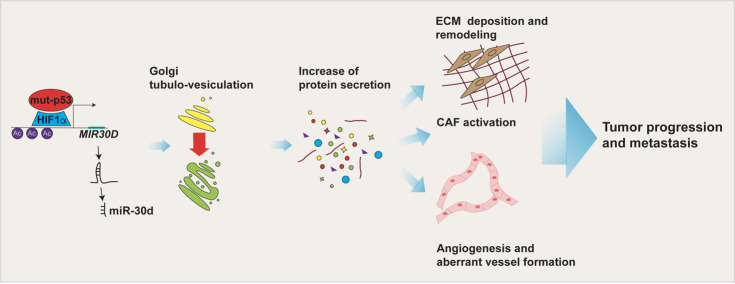
FIGURE 1: Impact of the mut-p53 secretome on the TME. mut-p53 in cooperation with HIF1α is able to bind *MIR30D* promoter and activate the expression of miR-30d. By targeting the diacylglycerol kinase ζ (DGKZ), miR-30d fosters diacylglycerol DAG signaling in the Golgi Apparatus, causing morphological and functional alterations (i.e. Golgi tubulo-vesiculation), responsible for the increase in total protein secretion. The mut-p53 driven secretome, including soluble factors and extracellular matrix (ECM) components, induces tumor cell invasion and migration, angiogenesis, alteration of mechanical properties of the ECM and fibroblasts activation. Altogether these TME features sustain tumor progression and metastasis.

miR-30d belongs to the miR-30 family of microRNAs, which in humans consists of five members (miR-30a, -30b, -30c, -30d and -30e) sharing a common seed sequence, previously described as either tumor suppressor or oncogenic miRNAs. Regarding miR-30d, it has been previously reported to be aberrantly expressed in different tumor contexts due to genomic amplification, to promote cancer cell migration/invasion and metastasis, and to be secreted by cancer cells.

The knowledge about miR-30d's contribution in influencing TME is still scattered. In human melanoma, miR-30d targets the GalNAc transferase GALNT7, affecting post-translational protein O-glycosylation and resulting in increased synthesis of the immunosuppressive cytokine IL-10, thereby inducing an immunosuppressive milieu and fostering metastasis. Strikingly, miR-30d has been reported to be secreted by both normal and cancer cells, to exert a role in intercellular communication between normal cells and has been proposed as a putative diagnostic/prognostic biomarker for some human tumors. Interestingly, it has been previously shown that miR-30d can specifically target the 3'UTR of p53 mRNA, leading to reduced expression of wt-p53. This notion is of great interest, since growing evidence supports the idea that loss of p53 in the TME, including cancer-associated fibroblasts, mesenchymal stem, myeloid suppressor cells and T cells, helps to promote tumorigenesis and causes immune escape. Thus, we can hypothesize that miR-30d oncogenic functions are achieved by multiple mechanisms, including wt-p53 downregulation in stromal cells, and that the presence of mut-p53 in cancer cells could contribute in rendering p53-null its microenvironment in a non-cell autonomous way. Of note, the effect of miR-30d on mut-p53 expression is very mild, most likely due to the high stability of mut-p53. Furthermore, it is conceivable that, once secreted by cancer cells in a mut-p53-dependent fashion, secreted miR-30d might exert its oncogenic functions also in receiving cells.

## MUT-p53 MODIFIES THE CELL SECRETORY MACHINERY

**Figure 2:**
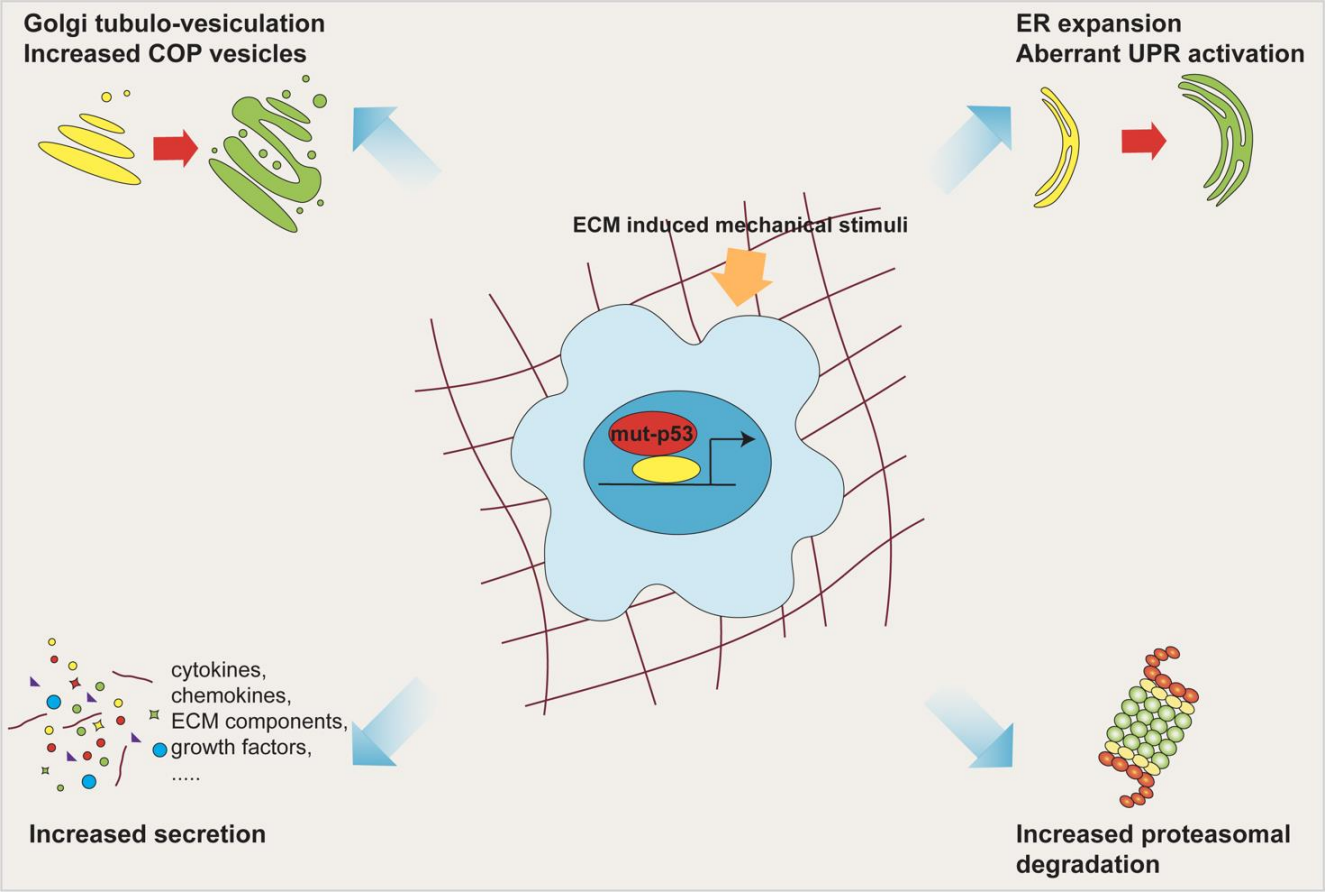
FIGURE 2: Mut-p53 modifies the cell secretory pathway. Mut-p53, via miR-30d, induces major structural alterations of the secretory pathway components, including ER enlargement, increase in number of COP vesicles, and tubulo-vesiculation of the Golgi apparatus. These morphologic alterations, together with overwhelming of ER folding capacity and enhancement of proteasomal protein degradation, cause functional modification of the whole secretory pathway, culminating in increased trafficking rate. All these features are fostered by mechanical stimulation deriving from the ECM.

In this work we found that mut-p53, via miR-30d, induces major structural alterations of secretory pathway components, resulting in enlargement of the endoplasmic reticulum (ER), increase in number of COP-I and COP-II vesicles, stabilization of microtubules and vesiculo-tubulation of the GA. These morphological alterations reflect in a functional modification, *i.e.*increased trafficking rate (**Figure 2**).

Downstream of mut-p53, we found two miR-30d targets whose inhibition may derange the secretory pathway. One of them, DGKZ, belongs to the diacylglycerol (DAG) kinase family, is known to regulate trafficking and secretion by multiple mechanisms. Downregulation of DGKZ increases local concentration of DAG at the Golgi membrane, facilitating vesicular transport and secretion by activating protein kinase PKD signaling. The second direct target of miR-30d is VPS26B, a component of the core retromer complex, which mediates the recycling of proteins during endosomal sorting; defects in retromer recycling may perturb GA dynamics and lead to aberrant secretion.

Typically, mammalian GA is composed by flattened cisternae, connected together to form a perinuclear ribbon; its localization and architecture are highly dynamic, and depend on input/output of membranes, interactions with the cytoskeleton, and on cellular demands. In different human cancers, the Golgi network has been found to be expanded and the ER-Golgi trafficking genes upregulated, suggesting that hijacking the secretory pathway to drive malignant secretion confers selective tumor advantages. In particular, our findings reveal that mut-p53, through miR-30d, causes the formation of tubular continuities across Golgi cisternae, and such alteration are known to promote rapid diffusion of cargoes within the Golgi, resulting in increased secretion.

In highly secretory cancer cells, overwhelming of ER folding capabilities associates with persistent ER stress. Recent evidence provided by our group and by others showed that mut-p53 regulates ER functions at multiple levels. Mut-p53 induces the UDPase ENTPD5, which favors the folding of N-glycosilated proteins, and alters the balance between the branches of the Unfoded Protein Respose (UPR), dampening the activity of IRE1α and PERK, while promoting ATF6 activation, ultimately resulting in increased cancer cell survival. Interestingly, mir-30d downregulates the levels of BiP/GRP78, one of the key regulators of the ER UPR, in prostate cancer cells. These data, coupled with the ability of mut-p53 to enhance proteasomal protein degradation, previously shown by our group, supports the hypothesis that mut-p53 acts as a regulator of secretory pathway homeostasis.

Nevertheless, several aspects of the impact of mut-p53 on the function of the GA and the secretory pathway remain unexplored. In fact, the GA is a crucial hub in the processes of intracellular trafficking, secretion and glycosylation, but also controls a wide variety of cellular processes including cytokinesis, apoptosis, cell migration, polarity, DNA repair and signal transduction. Chronic UPR and/or Golgi dysfunction, similar to those caused by mut-p53, might cause defects in protein glycosylation. Interestingly, the transport of proteins through GA cisternae (vesicular vs non-vesicular including tubules) promotes distinct glycosylation pathways, and mis-glycosylation of ECM components has been implicated in tumor promoting inflammation and immunosuppression, potentially disclosing another layer of complexity in the effect of mut-p53 on the crosstalk between cancer cells and TME.

Lastly, increase of the ER/Golgi trafficking can also favor transport of several transcription factors that require processing in the Golgi to be activated, such as ATF6, SREBPs and CREB3. Notably, activation of CREB3 alters breast cancer secretome, promoting aggressive phenotypes. The functional relevance of mut-p53 in these aspects is still unexplored and needs to be further investigated in the future.

## MUT-p53 AT THE CROSSROADS BETWEEN MICROENVIRONMENT AND CELL MECHANICS

In our work we found a novel mechanism by which mut-p53 physically reshapes the local environment by remodeling ECM composition and mechanical properties.

Interestingly, trafficking is under control of extracellular and intracellular forces and responds to aberrant mechanical cues, a condition frequently found in cancer. Notably, GA, being embedded in the actomyosin network, senses mechanical signals and responds by modulating its biochemical and structural properties; connection of the GA with F-actin via the GOLPH3/MYO18A bridge confers tensile forces required for efficient vesicles budding, and ROCK–Myosin II signaling controls secretion at the invading tumor edge in melanoma.

In addition, both mut-p53 and HIF1α are activated downstream to actomyosin dynamics induced by a rigid ECM. Consistently, elevated ECM stiffness also induces miR-30d expression and promotes cancer cell secretion. Thus, in tumors, mechanical stimuli may impact the secretory pathway not only by direct transmission of tensile forces but also by engaging the HIF1α/mut-p53/miR-30d program. In sum, this oncogenic axis establishes a feed-forward circuitry that both senses and generates mechanical cues, increasing cancer cell secretion and hence tumor aggressiveness.

